# Case Report: Use of Amniotic Microvesicles for Regenerative Medicine Treatment of a Mare With Chronic Endometritis

**DOI:** 10.3389/fvets.2020.00347

**Published:** 2020-06-17

**Authors:** Anna Lange-Consiglio, Federico Funghi, Carlo Cantile, Antonella Idda, Fausto Cremonesi, Pietro Riccaboni

**Affiliations:** ^1^Department of Veterinary Medicine (DIMEVET), Università degli Studi di Milano, Via dell'Università, Lodi, Italy; ^2^Independent Researcher, Grosseto, Italy; ^3^Department of Veterinary Science, Università di Pisa, Viale delle Piagge, Pisa, Italy

**Keywords:** biopsy, embryo, endometritis, mare, microRNAs, microvesicles, regenerative medicine

## Abstract

Chronic endometritis is an inflammation in the inner layer of uterine mucosa, with or without an infectious process, which affects the animal's fertility but not its general health. A variety of treatments has been adopted over the years but to date, no effective cures have been able to renew the injured tissue. Since the defects in the fetal-maternal communication are caused by degenerative changes due to chronic endometrial inflammation, our working hypothesis was a new approach to this disease by the regenerative medicine using amniotic derived microvesicles (MVs) for their anti-inflammatory and regenerative effects. The MVs are responsible for horizontal transfer of genetic materials, including microRNA (miRNAs) that are involved in paracrine communication between origin cells and target cells. Thus, intrauterine MV infusion may be beneficial in degenerative chronic endometritis and in the fetal–maternal talk. The selected mare was an 11-year-old Friesian, with a history of failed pregnancies despite numerous insemination attempts. Punctual and evident heats characterized the reproductive history, but no insemination attempts had been made for many years. The first (failed) insemination was when the mare was 9-years-old. In the next two reproductive seasons, other attempts were made at regular intervals but none was successful. After a final insemination attempt using a stallion of proven fertility, the collection of an 8-day old embryo suggested that the mare was affected by implantation failure related to endometritis. The mare was treated with two cycles of intrauterine administration of amniotic-derived MVs. The success of the intrauterine administration of MVs was demonstrated by an improvement in the classification of endometritis and in a successful artificial insemination (AI) with implantation of an embryo, as detected at day 14 and with a pregnancy that is still ongoing. Probably, MVs were able to restore the injured endometrium and re-establish the proper communication for a successful embryo implantation.

## Introduction

Fetal-maternal communication is an essential requirement for the recognition and maintenance of pregnancy. Early embryo loss during the pre-implantation phase is common in mammalian species and the pre-implantation phase is a particularly difficult stage in mares (1, 2). The communication between uterus and *conceptus*, essential for adhesion and implantation, occurs via paracrine signaling. Defects in the endometrial tissue could hinder this passage of information and lead to reduced fertility (3). Endometrial inflammations represent one of the causes of reproductive failure in the mare. The main consequence of endometritis is a uterine environment that is hostile for embryo survival and implantation, causing embryo death and abortion (6). Often, endometritis is due to infection by sexually or non-sexually transmitted pathogens, but in a subpopulation of susceptible mares a particular persistent mating induced endometritis (PMIE) also occurs (6). Chronic endometritis is an inflammation in the inner layer of uterine mucosa, with or without an infectious process, which affects the animal's fertility but not its general health. Chronic endometritis could develop because of unresolved PMIE or as a result of reduced intrinsic uterine defenses, leading to uncontrolled proliferation of resident microflora causing persistent inflammation (7). Chronic endometritis is one of the most frequent causes of infertility, early embryo death, and embryo death in broodmares (4, 5). Another chronic degenerative condition of multifactorial origin, such as age related changes to vascularity and tissue, vascular elastosis, tissue hypoxia, alterations in tissue remodeling, is called endometrosis (8). A variety of treatments have been adopted over the years, most of which are based on empirical practice and are frequently controversial (9).

Despite the wide range of therapies proposed, their efficacy has been called into question over the heterogeneity of responses (10, 11) and, to date, no effective treatments have been able to renew the injured tissue or to stop this process. Recently, stem cell treatment has been proposed as an alternative therapy of uterine disease (12, 12–14, 14, 15). Since the uterine glandular secretion is considered essential for fetal–maternal communication and embryo implantation, this report proposes a novel therapeutic approach based on endometrium regeneration and on restoration of paracrine fetal–maternal talk by the use of amniotic-derived MVs that represent the mechanism of paracrine action of mesenchymal cells.

## Case Details

### Signalment, History, and Clinical Findings

**Figure 1:**
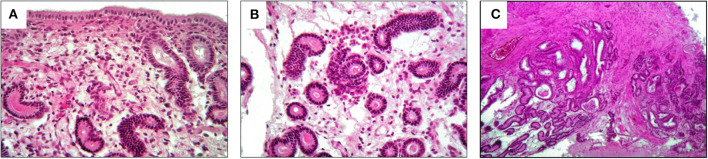
Histological results of pre-treatment biopsy. They show a category IIB endometritis. **(A)** Lymphocytes and plasma cells in the stratum compactum and spongiosum; **(B)** periglandular inflammatory clusters; **(C)** fibrotic glandular nests.

The selected mare was an 11-year-old Friesian, with a history of failed pregnancies despite numerous insemination attempts. The owner reported that the animal was used for riding and competition in the past but had recently been turned out to pasture. No previous pathologies were reported. The reproductive history was characterized by punctual and evident heats, but no insemination attempts had been made for many years. The first (failed) insemination was when the mare was 9-years-old. In the next two reproductive seasons, eight other attempts (four times in each season) both naturally and artificially were made but none was successful. Gynecological examination and instrumental investigations were normal and there was no relevant history. Visual examination of external genitalia focused on the perineal conformation to assess the level between the anus and vulva, paying attention to previous lacerations or Caslick interventions. In addition, a manual check of the integrity of the vestibular-vaginal sphincter was performed to exclude secondary infections due to pneumovagina or fecal contamination. The inspection of vagina and cervix was performed with a sterile tubular speculum to detect alterations, such as purulent drainage, cervical lesions, or urine stagnation that might indicate endometritis. The vestibule-vaginal features were also observed to detect the stage of the estrus cycle. A slight increase in tone and consistency of the uterus and, particularly of the cervix was detected. Lastly, endometrium biopsies were performed with a pair of 60 cm alligator forceps, taking a sample from the dorsal wall of the uterine body. The aim of the biopsy was to assess the starting condition of the mare and the results revealed a degenerative chronic category IIB endometritis. The histological sample showed a continuous endometrial epithelium composed of columnar cells sometimes raised in folds. Infiltration of lymphocytes and plasma cells was observed in the stratum compactum and spongiosum (Figure 1A) and as periglandular inflammatory clusters (Figure 1B). Inflammatory cells were accompained with diffuse edema and mild dilation of lymphatic vessels. Endometrial glands were mildly reduced in number with accumulation of eosinophilic secretion and some fibrotic glandular nests were also observed (Figure 1C).

To support the assumption that the failed inseminations were related to the pre-implantation phase and to exclude issues with conception, an insemination test (ninth attempt of insemination) was performed with the cryopreserved semen of a stallion of proven fertility. The collection of an 8-day old embryo confirmed that the issue was due to an inadequate fetal–maternal communication that would hinder a successful implantation.

## Treatment and Outcome

The therapeutic protocol proposed to treat this case was the intrauterine administration of MVs obtained by *in vitro* cultured of amniotic derived cells (AMCs). This protocol was approved by the Ethical Committee of the University of Milan (OPBA_118_2017). The AMCs were obtained at birth from the allanto-amniotic membrane of three broodmares. Samples were transported at 4°C in NaCl 0.9% solution supplemented with 4 mg/mL di amphotericin B (Euroclone), 100 UI/mL di penicillin, 100 mg/mL di streptomycin (Euroclone), and handled in laboratory within 12 h. The isolation method was previously described by Lange Consiglio et al. (16). Microvesicles isolation was performed following the protocol described by Perrini et al. (17).

**Figure 2:**
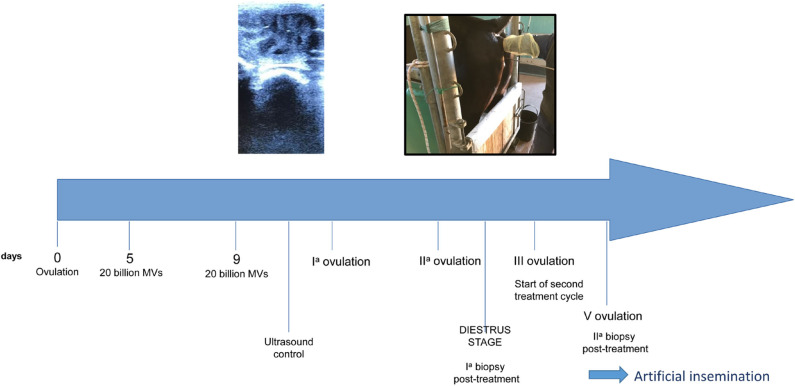
Schematic of treatment protocol.

**Figure 3:**
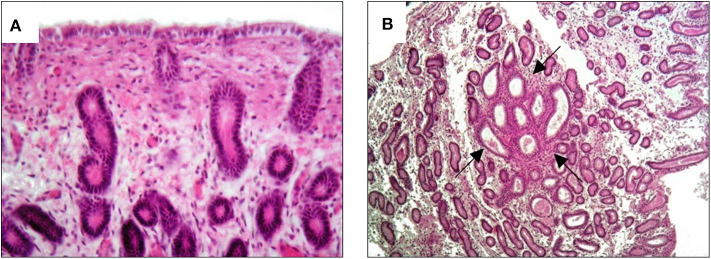
Histological results of biopsy after the first MV administration. An improvement to category IIA endometritis is showed. **(A)** Slight hemorrhage and edema in the stratum spongiosum with minimal inflammatory infiltration; **(B)** quantitatively regular glandular component with occasional fibrotic glandular nests (arrows).

The MV administration protocol was developed in our Reproduction laboratory and the schematic is shown in Figure 2. The protocol started on the ovulation day (day 0), which was detected by trans-rectal ultrasound and clinical examination. The first treatment was performed on day 5 with an intrauterine administration of 20 billion MVs diluted in 50 ml of NaCl 0.9%. A second administration was made on day 9. Thereafter, the mare was monitored every 48 h with ultrasound to detect the first and the second post-treatment ovulation. On the 10th day after the second post-treatment ovulation, during diestrum, a biopsy sample was collected. A second treatment cycle, with intrauterine MV administration at days 5 and 9 post-ovulation, was performed during the subsequent ovulation (third ovulation from the first administration). After two consecutive ovulations, a second biopsy was collected. The histologic analysis showed that after the first cycle of treatment, the endometritis was classified as category IIA and it was characterized by a reduction of inflammatory infiltration with residual microhemorrhages and edema in the stratum spongiosum (Figure 3A) and occasional fibrotic glandular nests (Figure 3B).

These features resulted from the previous inflammatory changes in the endometrium.

**Figure 4:**
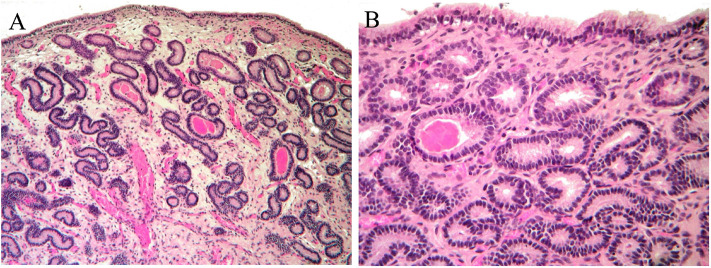
Histological findings of biopsy after the second MV administration. An improvement to I/IIA category endometritis is showed. **(A)** Normal glandular component and mild edema in the stratum spongiosum; **(B)** normal endometrial epithelium. Occasional accumulation of eosinophilic material in a few glands.

Moreover, the histologic analysis of the sample collected after the second cycle of treatment showed an additional improvement to I/IIA endometritis. Endometrial biopsy showed a normal glandular component with residual edema in the stratum spongiosum (Figure 4A) and some luminal distension containing eosinophilic material (Figure 4B).

**Figure 5:**
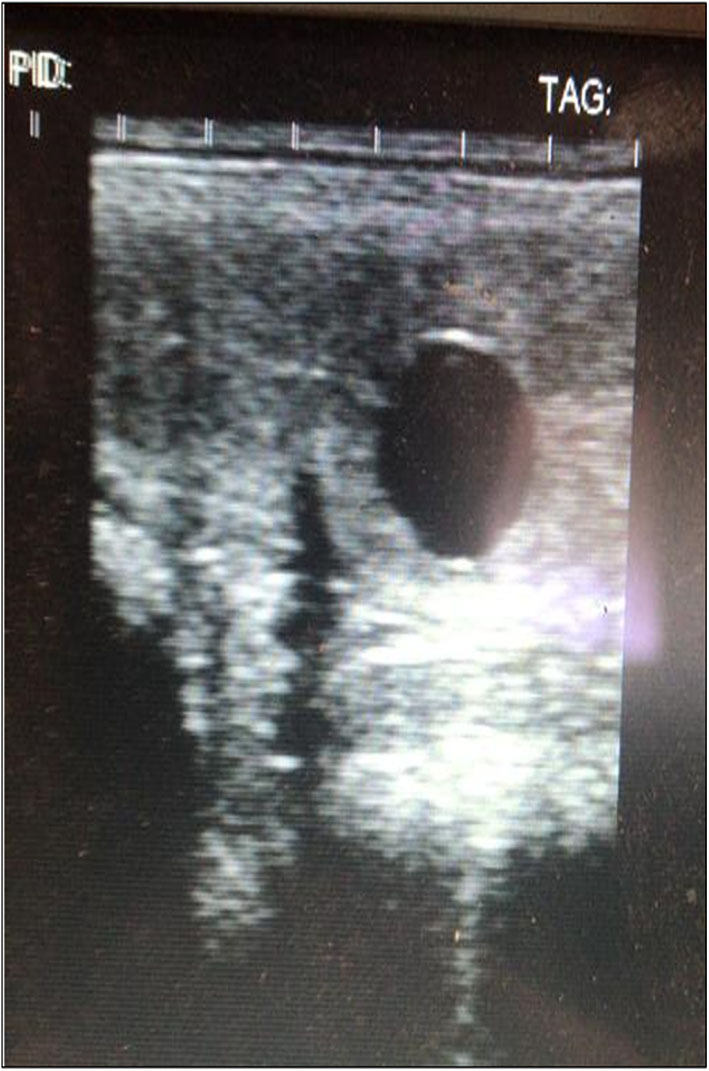
Ultrasound results. They show a 14-day embryo detected after two cycles of MV administration.

Given the positive improvements in the control biopsies and at the request of the owner, ovulation was induced with Buserelin (Receptal, MSD Animal Health, Segrate, Mi, Italy) and AI was performed using cryopreserved sperm. No inflammatory fluid retention was observed in the uterine lumen after this intervention, similarly to what was detected during all the ultrasound clinical evaluation performed in the past. On the 14th day after insemination, the first ultrasound check identified an embryonic vesicle and, thus, the presence of a pregnancy that is still ongoing (Figure 5). The mare did not develop delayed uterine clearance after embryo collection or in the past.

## Discussion and Conclusions

When mesenchymal stromal cells (MSCs) were first used in regenerative medicine, their therapeutic properties were attributed to a direct action of differentiation at the site of damage but it has since been shown that the main mechanism of action is paracrine signaling (18). This communication occurs via a cellular secretome that contain soluble factors, such as cytokines, chemokines, neurotransmitters, and hormones (19), but also insoluble factors represented by lipid bilayer-delimited particles called microvesicles (MVs). Literature shows that the beneficial effects obtained with secretome are equivalent to those obtained using the cells from which they are derived (16, 20, 21).

In recent years, the regenerative mechanism of MSCs has been focused on MV action responsible for horizontal transfer of cytoplasmic content including miRNAs (22). In general, miRNAs have a key role in cell development, proliferation, differentiation, apoptosis, and signal transduction (23). In the reproductive system of mammals, MVs have a key role in follicular growth and oocyte maturation (24–26). In horses, profiles of MV miRNAs in follicular fluid vary with age and are associated with fertility (24, 27). Paracrine signaling between the conceptus and endometrium is important for fertility and is likely controlled by miRNAs. A failure in this communication leads to implantation failure. Day 8 equine embryos are believed to secrete MVs that can modulate the function of the oviductal epithelium by the transfer of early pregnancy factors, such as HSP10 and miRNA (28). MVs, which target biological pathways relevant for embryo implantation, are also secreted from the endometrial epithelium (29). This evidence supports our initial hypothesis about regenerative medicine with MVs, which may be able to restore both the injured endometrium and re-establish the proper communication for a successful embryo implantation.

This is the first report discussing the case of a mare affected by chronic degenerative endometritis and with a history of failed inseminations, which appeared to have been resolved following intrauterine administration of MVs secreted *in vitro* by AMCs.

In this mare, infertility was caused by a defect in the implantation phase, and not related to conception, as suggested by the ability to form an 8-day embryo during pre-treatment tests. The success of the intrauterine administration of MVs was demonstrated by post-treatment histologic results that showed an improvement in the classification of endometritis. At the end of treatment, the mare was classified as almost normal, and 14 day after AI an embryo was successfully detected by ultrasound and the pregnancy is still ongoing.

Of course, any of the MVs cargo may have contributed to an improvement in the pathological picture of this mare, but our attention has been mainly focused on miRNAs that are non-coding RNA molecules of about 22 nucleotides in length that induce gene silencing through translation repression or degradation of the target molecule (30). In a comparative study on the miRNA cargo between equine amniotic mesenchymal cells and their respective MVs, Lange Consiglio et al. (22), extracted and deeply sequenced small RNAs followed by miRNA in silico detection. The results showed that there are 146 miRNAs differentially expressed (DE) between AMCs and MVs. Among the known DE miRNAs, 17 showed higher expression in MVs confirming that there is a compartmentalization of miRNAs in MVs and that the miRNA cargo of MVs is different compared to that of amniotic origin cells. In addition, this report (22) show that in amniotic derived MVs there are three specific miRNAs that have also been studied in other species, and are mainly involved in modulation of the inflammatory response. These miRNAs are: miR-223, which inhibits the expression of IL-6, IL-1b, and TNFα by macrophages (31); miR-150, which decreases the production of some inflammatory cytokines such as IL-2 and TNFα (32); and miR-126 which promotes angiogenesis and blocks inflammation of endothelial cells (33).

In the physiopathologic mechanisms of mare endometrosis, such as in other inflamed tissues, pro-inflammatory cytokines, growth factors, and chemokines are involved acting on fibroblast and other cells, and influencing extra-cellular matrix deposition and tissue fibrosis (34–36). The action of the miRNAs, probably, was that of down-regulating the expression of pro-inflammatory molecules, reducing the apoptosis rate, increasing endometrial cell proliferation, and restoring the anti-inflammatory level of some cytokines, such as transforming growth factor-β, as demonstrated in some *in vitro* studies (17, 37). In this context, it could therefore be assumed that, in this mare, the action of MVs and the miRNAs contained in them induced tissue regeneration with a return to the original histologic characteristics and, consequently, to the recovery of normal endometrial function. This probably restored the conditions that are the basis of maternal-embryonic paracrine communication, which are necessary for normal development and implantation.

## Data Availability Statement

All datasets generated for this study are included in the article/supplementary material.

## Ethics Statement

This study was approved by the University of Milan Ethics Committee (Protocol Number 118-2017). All procedures were conducted following standard veterinary practice and in accordance with 2010/63 EU directive on animal protection. The informed client consent was obtained for collection placenta at term of horse pregnancies and for horse treatments.

## Author Contributions

FC and AL-C designed the study. AL-C and AI performed AMC isolation and MV preparation. FC and FF enrolled animal, performed ultrasonography evaluation, biopsies, MV administration. AI pregnancy diagnosis. CC and PR performed biopsy analyses. AL-C and FC evaluated the results of biopsies. AL-C wrote the manuscript. All co-authors provided useful comment on the manuscript.

## Conflict of Interest

The authors declare that the research was conducted in the absence of any commercial or financial relationships that could be construed as a potential conflict of interest.

## Abbreviations

AI: artificial insemination
AMCs: amniotic mesenchymal cells
AMC-MVs: MVs obtained from AMC
MVs: microvesicles
miRNA: microRNA
MSCs: mesenchymal stromal cells
MVs: microvesicles
PMIE: persistent mating induced endometritis.
